# BMP signaling alters aquaporin-4 expression in the mouse cerebral cortex

**DOI:** 10.1038/s41598-021-89997-5

**Published:** 2021-05-18

**Authors:** Kazuya Morita, Naoyuki Matsumoto, Kengo Saito, Toshihide Hamabe-Horiike, Keishi Mizuguchi, Yohei Shinmyo, Hiroshi Kawasaki

**Affiliations:** 1 Department of Medical Neuroscience, Graduate School of Medical Sciences, Kanazawa University, Takara-machi 13-1, Kanazawa, Ishikawa 920-8640 Japan; 2 Medical Research Training Program, School of Medicine, Kanazawa University, Kanazawa, Ishikawa 920-8640 Japan

**Keywords:** Development of the nervous system, Molecular neuroscience

## Abstract

Aquaporin-4 (AQP4) is a predominant water channel expressed in astrocytes in the mammalian brain. AQP4 is crucial for the regulation of homeostatic water movement across the blood–brain barrier (BBB). Although the molecular mechanisms regulating AQP4 levels in the cerebral cortex under pathological conditions have been intensively investigated, those under normal physiological conditions are not fully understood. Here we demonstrate that AQP4 is selectively expressed in astrocytes in the mouse cerebral cortex during development. BMP signaling was preferentially activated in AQP4-positive astrocytes. Furthermore, activation of BMP signaling by in utero electroporation markedly increased AQP4 levels in the cerebral cortex, and inhibition of BMP signaling strongly suppressed them. These results indicate that BMP signaling alters AQP4 levels in the mouse cerebral cortex during development.

## Introduction

Aquaporin-4 (AQP4) is a predominant water channel in the mammalian brain and is mainly expressed at the endfeet of astrocytes^1–3^. In the cerebral cortex, AQP4 plays crucial roles in controlling water homeostasis. AQP4 regulates water movement across the blood–brain barrier (BBB), which restricts large polar substances and potentially neurotoxic compounds in circulation from passively diffusing into the brain^4,5^. Changes in AQP4 expression are often associated with various diseases such as epilepsy, edema and glioblastoma^6–10^. Thus, uncovering the molecular mechanisms regulating the expression of AQP4 is an important research topic. However, our understanding of the mechanisms of regulating AQP4 expression in the cerebral cortex is still rudimentary.

Previous reports have investigated the regulation of AQP4 expression in the context of inflammation in the mouse brain^11^. The induction of oxidative stress activates the p38 mitogen-activated protein kinase (MAPK) pathway, inducing transcriptional activation of *AQP4*^12^. Activation of the cysteinyl leukotriene receptor 2 induces AQP4 upregulation through the p38 and ERK signaling pathways in astrocytes^13^. In contrast to the regulation of AQP4 expression under pathological conditions, the mechanisms regulating AQP4 expression under normal physiological conditions in the cerebral cortex are not fully understood.

Bone morphogenetic proteins (BMPs), members of the TGF-β family of signaling molecules, have numerous functions in the brain^14–18^. Here, we show that BMP signaling was activated in astrocytes in the cerebral cortex. Activation of BMP signaling increased AQP4 levels in astrocytes in the mouse cerebral cortex in vivo*.* Consistently, inhibition of BMP signaling decreased AQP4 levels in astrocytes. Taken together with a previous study showing that activation of BMP signaling increased AQP4 expression in cultured astrocytes^19^, these results clearly indicate that BMP signaling changes AQP4 levels in astrocytes under normal physiological conditions.

## Results

### Expression patterns of AQP4 in the developing mouse cerebral cortex

**Figure 1:**
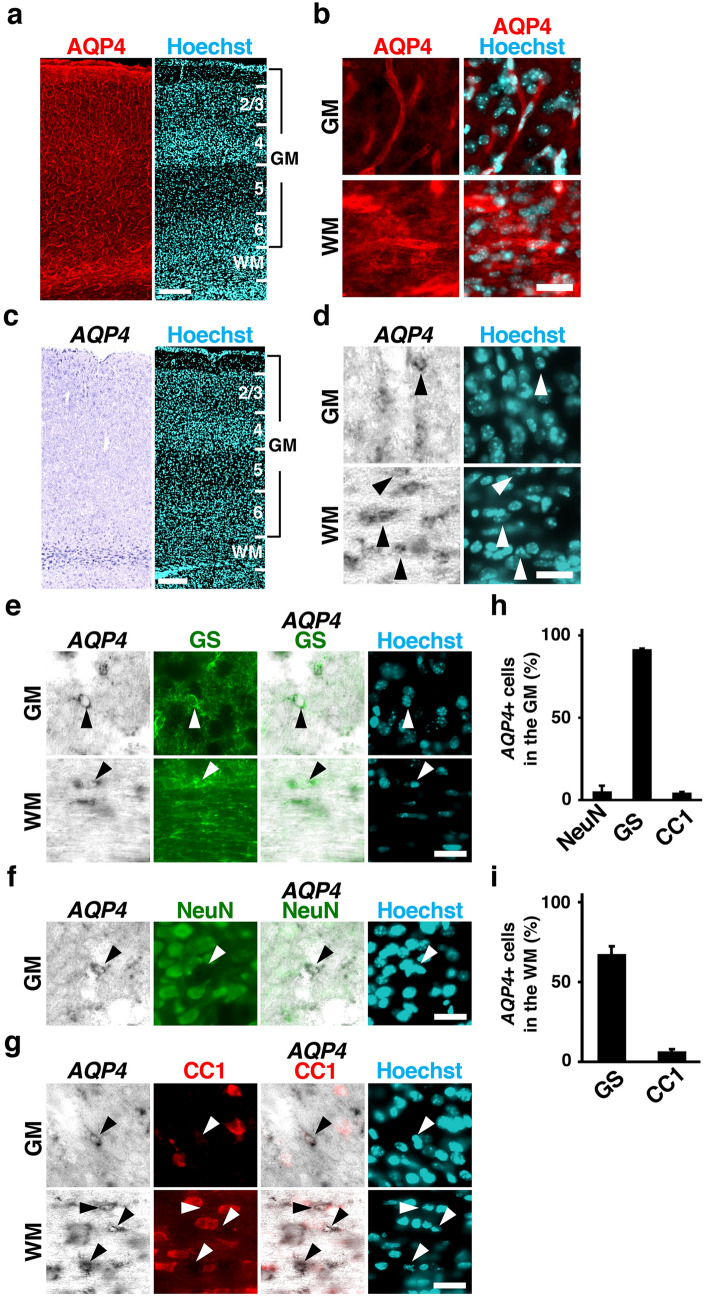
Expression patterns of AQP4 in the developing mouse cerebral cortex. (**a**,**b**) Sections of the mouse cerebral cortex at P16 were subjected to AQP4 immunohistochemistry and Hoechst 33342 staining. Low magnification images (**a**) and high magnification images (**b**) corresponding to the gray matter (GM) and the white matter (WM) are shown. AQP4 was expressed throughout the developing mouse cerebral cortex. The expression level of AQP4 was higher in the WM than in the GM. (**c**,**d**) Sections of the mouse cerebral cortex at P16 were subjected to in situ hybridization for *AQP4* and Hoechst 33342 staining. Low magnification images (**c**) and high magnification images (**d**) corresponding to the GM and the WM are shown. *AQP4* mRNA was preferentially distributed in the WM but was also observed in the GM. (**e–g**) Sections of the mouse cerebral cortex at P16 were subjected to in situ hybridization for *AQP4* and immunohistochemistry for either GS (**e**), NeuN (**f**) or CC1 (**g**). High-magnification images of the GM and the WM are shown. *AQP4* was preferentially expressed in GS-positive cells (**e**, arrowheads), while *AQP4* signals were not colocalized with NeuN-positive cells or CC1-positive cells (**f** and **g**, arrowheads). (**h**,**i**) The percentages of *AQP4*-positive cells co-expressing NeuN, GS, and CC1 in the GM (**h**) and the WM (**i**). n = 3 animals for each condition. Bars represent mean ± SD. Numbers indicate the corresponding layers in the cerebral cortex. GM, gray matter; WM, white matter. Scale bars 200 µm (**a**,**c**) and 25 µm (**b**,**d–g**).

To investigate the expression patterns of AQP4, we performed immunohistochemistry for AQP4 using the mouse cerebral cortex at postnatal day 16 (P16) (Fig. 1a,b). AQP4 was expressed throughout the cortex, though the expression levels of AQP4 were higher in the white matter (WM) than that in the gray matter (GM). Consistent with a previous study showing that AQP4 is distributed at the endfeet of astrocytes attached to blood vessels^20^, vascular structures were labeled with AQP4 immunostaining (Fig. 1b). Next, we investigated the distribution patterns of AQP4-positive cells using in situ hybridization for *AQP4*. Consistent with the results of AQP4 immunostaining, *AQP4* mRNA was preferentially distributed in the WM but was also observed in the GM (Fig. 1c,d).

Although it has been reported that AQP4 is expressed only in astrocytes in the cerebral cortex of adult mice^1,2^, it remained unknown which cell types express AQP4 during development. To examine which cell types express AQP4 in the mouse cerebral cortex during development, we combined in situ hybridization for *AQP4* and immunohistochemistry for either the astrocyte marker glutamine synthetase (GS), the neuron marker NeuN or the oligodendrocyte marker CC1 (Fig. 1e–g). Consistent with previous reports using the adult brain^1,2^, *AQP4* was preferentially expressed in GS-positive cells in the mouse cerebral cortex at P16, while *AQP4* signals were not co-localized with NeuN-positive cells or CC1-positive cells (Fig. 1h,i) (GM; NeuN, 5 ± 3%; GS, 92 ± 0.5%; CC1, 5 ± 0.3%) (WM; NeuN, 0%; GS, 67 ± 5%; CC1, 7 ± 1%). These results suggest that AQP4 is mainly expressed in astrocytes in the developing mouse cerebral cortex.

### BMP signaling is activated in AQP4-positive astrocytes in the developing mouse cerebral cortex

**Figure 2:**
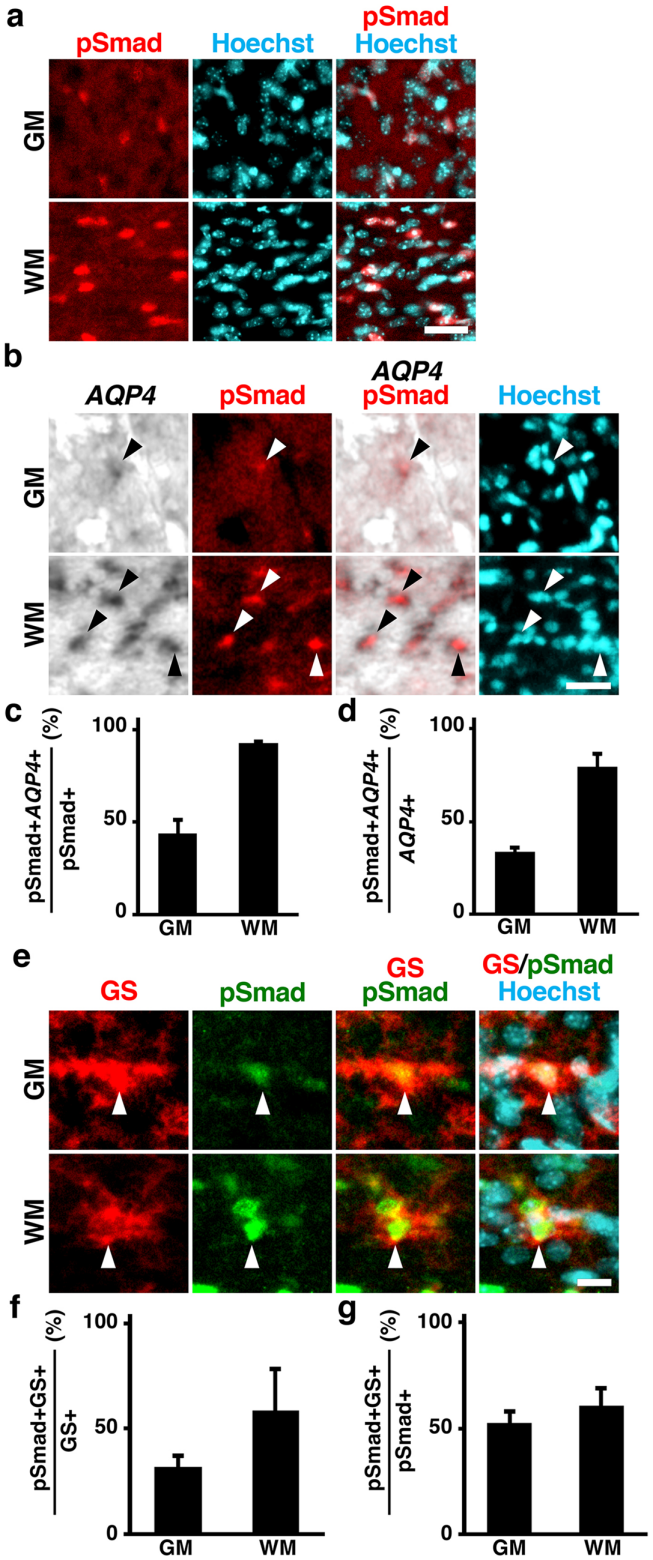
BMP signaling is activated in AQP4-positive astrocytes in the developing mouse cerebral cortex. (**a**) Sections of the mouse cerebral cortex at P16 were subjected to immunohistochemistry for pSmad and Hoechst 33342 staining. pSmad signals were more predominant in the WM compared with the GM. (**b**) Sections were subjected to immunohistochemistry for pSmad and in situ hybridization for *AQP4*. High magnification images are shown. Many *AQP4*-positive astrocytes were also labeled with pSmad (arrowheads). (**c**) The percentages of pSmad-positive cells co-expressing *AQP4* in the GM and the WM. (**d**) The percentages of *AQP4*-positive cells co-labeled with pSmad in the GM and the WM. (**e**) Sections of the mouse cerebral cortex at P16 were subjected to immunohistochemistry for pSmad and GS. High magnification images are shown. pSmad signals were observed in GS-positive cells (arrowheads). (**f**) The percentages of GS-positive cells co-labeled with pSmad in the GM and the WM. (**g**) The percentages of pSmad-positive cells co-expressing GS in the GM and the WM. GM, gray matter; WM, white matter. n = 3 animals for each condition. Bars represent mean ± SD. Scale bars 25 μm (**a**,**b**) and 10 µm (**e**).

We next investigated the molecular mechanisms regulating AQP4 levels during cortical development. We performed immunohistochemistry for phospho-Smad1/5/8 (pSmad) using cortical sections of mice at P16. Consistent with a previous report^21^, pSmad, which is induced by the activation of BMP signaling, was observed in the mouse cerebral cortex at P16, more predominantly in the WM (Fig. 2a).

Because both *AQP4* and pSmad were predominantly distributed in the WM, we hypothesized that BMP signaling affects AQP4 levels. To test this, we performed immunohistochemistry for pSmad and in situ hybridization for *AQP4* and found that most pSmad-positive cells in the WM expressed *AQP4* (92 ± 1%) (Fig. 2b,c). Furthermore, most of the *AQP4*-positive cells in the WM were positive for pSmad (79 ± 6%) (Fig. 2d). Consistent with our observation, the percentage of pSmad-positive cells co-expressing *AQP4* and that of *AQP4*-positive cells co-labeled with pSmad were smaller in the GM (Fig. 2c,d).

To examine whether pSmad is distributed in astrocytes, we performed immunohistochemistry for pSmad and GS. We found that pSmad was mainly observed in GS-positive cells in the WM (59 ± 20%) (Fig. 2e,f). The percentage of GS-positive cells co-labeled with pSmad was smaller in the GM (Fig. 2e,f). More than half of pSmad-positive cells expressed GS in the GM and the WM (GM, 53 ± 6%; WM, 61 ± 8%) (Fig. 2g). These results suggest that BMP signaling is activated in *AQP4*-positive astrocytes, especially in the WM, during development. Consistent with our observations, a previous study demonstrated that BMP receptors were preferentially expressed in astrocytes in the developing mouse cerebral cortex^22^.

### Activation of BMP signaling increases AQP4 levels in the mouse cerebral cortex

To directly test whether BMP signaling alters AQP4 levels, we stimulated BMP signaling by introducing BMP7 into the mouse cerebral cortex using in utero electroporation (IUE) at embryonic day 14 (E14) and examined AQP4 expression at P16. Previous studies and the Allen Brain Atlas (https://portal.brain-map.org/) have shown that BMP family members such as BMP3 and BMP7 are expressed in the cerebral cortex during development^23,24^. Because it was reported that BMP3 does not have the ability to activate Smad1/5/8 or to induce bone and cartilage tissues in vivo, and because most BMP ligands share Smad1/5/8 as downstream mediators^25^, here we utilized BMP7 to activate BMP signaling. In situ hybridization for *BMP7* confirmed that *BMP7* mRNA was indeed produced as a result of introducing BMP7-expressing plasmids into the mouse cerebral cortex (Supplementary Fig. S1a). Furthermore, immunostaining for pSmad demonstrated that BMP signaling was strongly activated by the introduction of BMP7-expressing plasmids (Supplementary Fig. S1b). In addition, it should be noted that the layer structure of the cerebral cortex was not affected by BMP7 electroporation (Supplementary Fig. S2).

**Figure 3:**
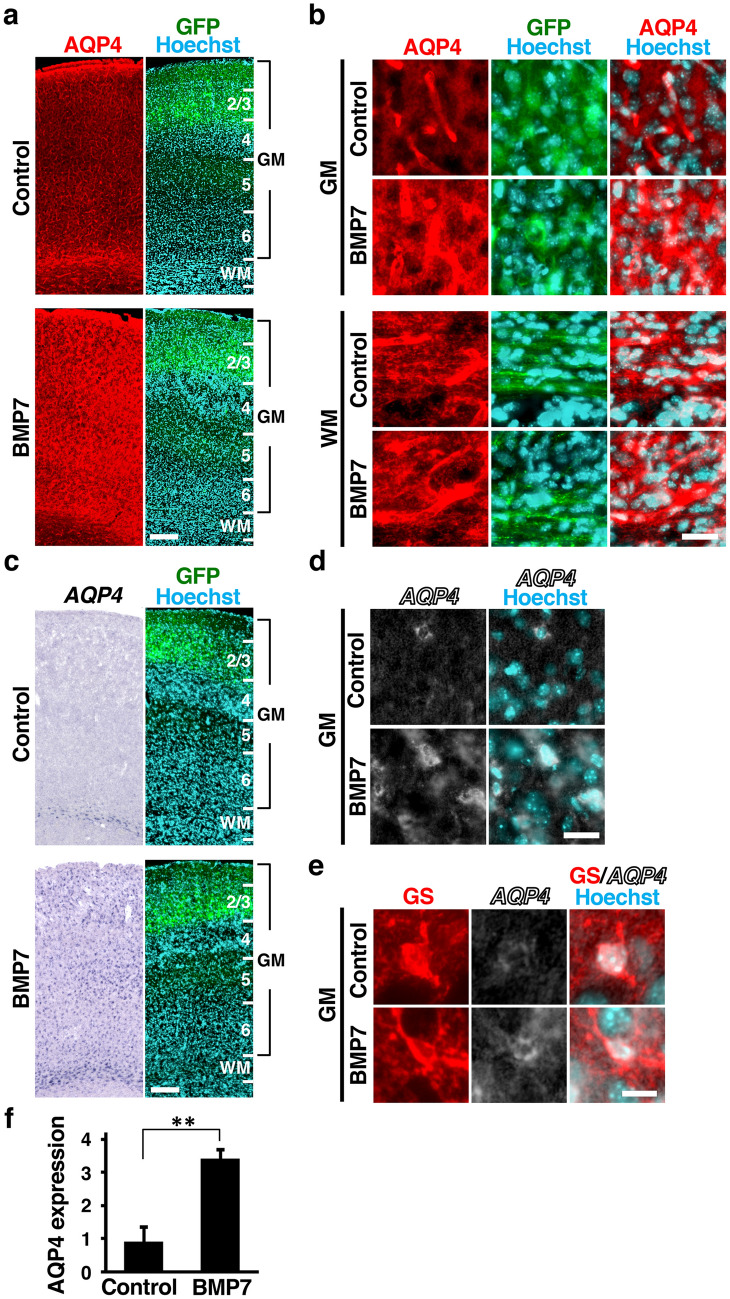
Activation of BMP signaling increases AQP4 levels in the mouse cerebral cortex. CAG-EGFP plus either pCAG-BMP7 or pCAG control vector was electroporated at E14, and the brains were dissected at P16. (**a**,**b**) Coronal sections stained with anti-AQP4 antibody, anti-GFP antibody and Hoechst 33342. Low magnification images (**a**) and high magnification images of the GM and the WM (**b**) are shown. The immunoreactivity of AQP4 was markedly increased by activation of BMP signaling. (**c**,**d**) Sections were subjected to in situ hybridization for *AQP4* and Hoechst 33342 staining. Low magnification images (**c**) and high magnification images (**d**) are shown. Activation of BMP signaling significantly increased mRNA expression levels of *AQP4*. (**e**) Sections were subjected to in situ hybridization for *AQP4* and immunohistochemistry for GS. High magnification images are shown. Activation of BMP signaling increased mRNA expression levels of *AQP4* in GS-positive cells. (**f**) Quantification of AQP4 immunoreactivity in the cerebral cortex. Activation of BMP signaling significantly increased AQP4 immunoreactivity. n = 3 animals for each condition. Bars represent mean ± SD. **p < 0.01, Student’s t-test. Numbers indicate the corresponding layers in the cerebral cortex. GM, gray matter; WM, white matter. Scale bars 200 µm (**a**,**c**), 25 µm (**b**,**d**) and 10 µm (**e**).

We next performed immunohistochemistry and in situ hybridization for *AQP4* and found that the activation of BMP signaling markedly increased both AQP4 protein and *AQP4* mRNA in the cerebral cortex (Fig. 3a–d). The increase of AQP4 was clearer in the GM than in the WM, presumably because AQP4 expression in the GM of the control brain were lower than those in the WM (Fig. 3a,c). We performed in situ hybridization for *AQP4* and immunohistochemistry for GS and found that *AQP4* mRNA was increased in GS-positive cells (Fig. 3e). Our quantification of the intensity of AQP4 immunoreactivity showed that activation of BMP signaling significantly increased AQP4 protein in the cerebral cortex (control, 0.92 ± 0.43; BMP7, 3.41 ± 0.26; p < 0.01, Student’s t-test) (Fig. 3f). Taken together with a previous study showing that activation of BMP signaling increased AQP4 expression in cultured astrocytes^19^, these results indicate that activation of BMP signaling is sufficient for increasing AQP4 levels in astrocytes in the cerebral cortex.

### Inhibition of BMP signaling reduces AQP4 levels in the mouse cerebral cortex

**Figure 4:**
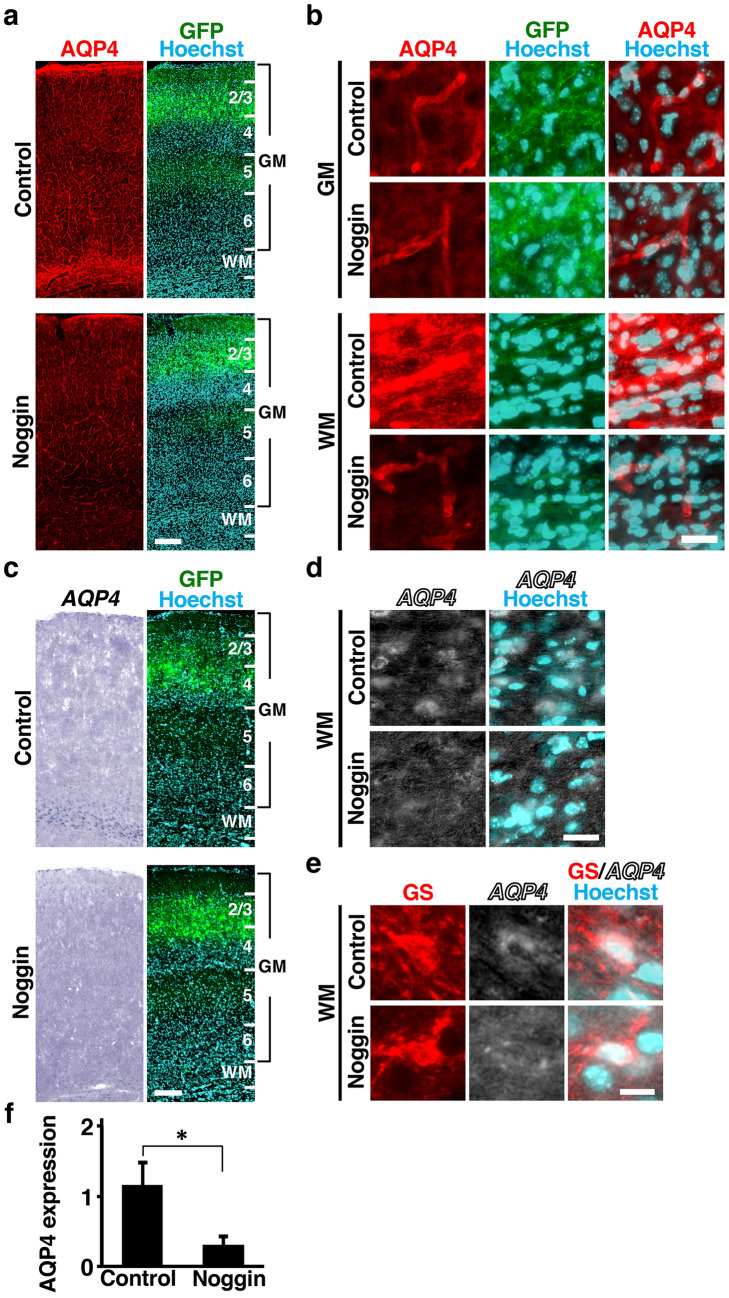
Inhibition of BMP signaling reduces AQP4 levels in the mouse cerebral cortex. pCAG-EGFP plus either pCAG-noggin or pCAG control vector was electroporated at E14, and the brains were dissected at P16. (**a**,**b**) Coronal sections stained with anti-AQP4 antibody, anti-GFP antibody and Hoechst 33342. Low magnification images (**a**) and high magnification images (**b**) of the GM and the WM are shown. The immunoreactivity of AQP4 in the WM was markedly decreased by noggin. (**c**,**d**) Sections were subjected to in situ hybridization for *AQP4* and Hoechst 33342 staining. Low magnification images (**c**) and high magnification images of the WM (**d**) are shown. Noggin significantly decreased mRNA expression levels of *AQP4* in the WM. (**e**) Sections are subjected to in situ hybridization for *AQP4* and immunohistochemistry for GS. High magnification images of the WM are shown. (**f**) Quantification of AQP4 immunoreactivity in the WM. Inhibition of BMP signaling by noggin significantly decreased AQP4 immunoreactivity in the WM. n = 3 animals for each condition. Bars represent mean ± SD. *p < 0.05, Student’s t-test. Numbers indicate the corresponding layers in the cerebral cortex. GM, gray matter; WM, white matter. Scale bars 200 µm (**a**,**c**), 25 µm (**b**,**d**) and 10 µm (**e**).

We next examined whether endogenous BMP signaling is required for inducing AQP4 levels. To test this, we used noggin, which binds specifically to BMPs and antagonizes BMP signaling by blocking the interaction of BMPs with their receptors^26^. Noggin was introduced into the mouse cerebral cortex using IUE at E14, and AQP4 expression was examined at P16. Immunostaining showed that pSmad signals were markedly reduced by noggin (Supplementary Fig. S1c), suggesting that BMP signaling was inhibited by the introduction of noggin-expressing plasmids using IUE. In addition, the layer structure of the cerebral cortex was not affected by noggin electroporation (Supplementary Fig. S2). Immunohistochemistry and in situ hybridization consistently showed that noggin markedly reduced AQP4 levels (Fig. 4a–d). The reduction of AQP4 signals in the WM was obvious, whereas that in the GM was not clearly observable (Fig. 4a,c). This seems to be because AQP4 levels in the GM of the control cerebral cortex were lower than those in the WM. We performed in situ hybridization for *AQP4* and immunohistochemistry for GS and found that noggin significantly decreased *AQP4* mRNA in GS-positive cells in the WM (Fig. 4e). Our quantification for the intensity of AQP4 immunoreactivity showed that the inhibition of BMP signaling significantly decreased AQP4 protein levels in the WM (control, 1.16 ± 0.33; noggin, 0.30 ± 0.13; p < 0.05, Student’s t-test) (Fig. 4f).

To further confirm the importance of endogenous BMP signaling, we utilized a soluble dominant-negative BMP receptor (sBMPR) which lacks both transmembrane and serine/threonine kinase domains^27^. sBMPR was introduced into the mouse cerebral cortex using IUE at E14, and the cerebral cortex was examined at P16. Consistent with the results obtained using noggin, immunohistochemistry showed that sBMPR reduced AQP4 levels (Supplementary Fig. S3a,b), Our quantification showed that sBMPR significantly decreased AQP4 immunoreactivity in the WM (control, 0.85 ± 0.28; sBMPR, 0.4 ± 0.13; p < 0.05, Student’s t-test) (Supplementary Fig. S3c). These results demonstrate that endogenous BMP signaling is essential for the AQP4 levels found in astrocytes. Taken together, our findings indicate that BMP signaling increases AQP4 levels in the mouse cerebral cortex during development.

## Discussion

Our experiments demonstrated that BMP signaling was activated in astrocytes which expressed AQP4. Activation of BMP signaling increased AQP4 levels in astrocytes*.* Consistently, inhibition of BMP signaling decreased AQP4 levels in astrocytes in the cerebral cortex. Our findings indicate that BMP signaling increases AQP4 levels in the mouse cerebral cortex during development.

### The physiological regulation of AQP4 levels

Although previous studies have examined the regulatory mechanisms of AQP4 levels, most of the studies investigated these mechanisms under pathological conditions such as inflammatory responses, hepatic encephalopathy, epilepsy, brain edema and glioblastoma^6–11^. For example, the exposure of astrocytes to ammonium chloride, which is an in vitro model of hepatic encephalopathy, activates the p38 MAP kinase pathway, and as a result, AQP4 expression is upregulated^12^. Oxygen–glucose deprivation induces AQP4 upregulation in astrocytes through the p38 MAP kinase and ERK signaling pathways^13^. However, little was known about the regulatory mechanisms of AQP4 levels under normal physiological conditions. A previous pioneering study using cultured astrocytes reported that BMP activation increased the expression levels of AQP4 protein in astrocytes in vitro^19^. Our gain- and loss-of-function experiments clearly demonstrated that endogenous BMP signaling increases AQP4 levels in the mouse cerebral cortex in vivo during development. Because a previous study reported that BMP signaling promoted astrocyte maturation, increased AQP4 expression and downregulated EGF receptor expression^19^, it seems possible that activation of BMP signaling induced astrocyte maturation and, as a result, AQP4 expression was increased in the cerebral cortex.

BMP can activate both canonical and non-canonical intracellular signaling pathways. In the canonical BMP signaling pathway, activation of BMP receptors phosphorylates Smad1/5/8, which makes a complex with Smad4. Then, the complex translocates into the nucleus and regulates gene expression. In the non-canonical BMP signaling pathway, BMP activates several molecules, which are distinct from Smad, such as p38 MAP kinase, JNK, ERK, PI3 kinase and Cdc42. Because previous studies demonstrated that p38 MAP kinase regulates AQP4 expression downstream of ammonium chloride and oxygen–glucose deprivation, it seems plausible that p38 MAP kinase mediates the effect of BMP on AQP4 levels in the cerebral cortex. In addition, because our experiments showed that Smad1/5/8 was activated in AQP4-positive astrocytes, it remains possible that the canonical BMP signaling pathway is involved in AQP4 expression. Further investigation would be necessary in order to uncover the entire picture of the mechanisms underlying the physiological regulation of AQP4 levels.

Previous studies using IUE reported that radial migration of cortical neurons was inhibited by introducing either BMP or dominant-negative BMP receptors at E17.5 or P0, respectively^21,28^. In contrast, our experiments using BMP and noggin did not show obvious effects on the cortical layers at P16 (Supplementary Fig. S2). It seemed plausible that neuronal migration was delayed by activation or inhibition of BMP signaling but finished by P16.

### Possible roles of AQP4 in the brain

The importance of AQP4 in the brain has been investigated intensively. It was reported that AQP4 has an important role in water and waste clearance in the brain. The deletion of AQP4 expression resulted in the reduction of interstitial solute clearance, and in the accumulation of amyloid β, a peptide thought to be pathogenic in Alzheimer’s disease^29^. Other studies reported that AQP4 is involved in sleep architecture. A cohort study of adult humans showed that genetic variants in the *AQP4* genome are related to sleep quality^30^. Another study reported an *AQP4*-haplotype associated with distinct modulations of non-rapid eye movement (NREM) sleep^31^. It seems plausible that the regulation of AQP4 expression by BMP signaling, which we uncovered in this study, is involved in the regulation of water and waste clearance and sleep quality. It would be intriguing to examine whether BMP signaling alters water and solute clearance and sleep quality through regulating AQP4 expression.

The BBB is important for brain homeostasis because the BBB restricts large polar substances and potentially neurotoxic compounds in the circulation from passively diffusing into the brain^4,5^. Therefore, it is important to elucidate the mechanism underlying the structural and functional development of the BBB. AQP4 is one of the major functional components of the BBB and is crucial for water transport at the blood–brain interface^1–3,5^. Our data showed that BMP signaling is necessary and sufficient for the expression of AQP4 in vivo, raising the possibility that BMP signaling regulates the function of the BBB by modulating AQP4 expression. Interestingly, it was reported that the structure of the BBB is also regulated by BMP signaling^32^. Taken together, BMP signaling may regulate the BBB not only structurally but also functionally through regulating AQP4 expression.

## Methods

### Animals

ICR mice were purchased from SLC (Hamamatsu, Japan) and reared on a normal 12 h light/dark schedule. The day of conception and that of birth were counted as embryonic day 0 (E0) and postnatal day 0 (P0), respectively. All procedures were performed in accordance with protocols approved by the Animal Care Committee of Kanazawa University and with the ARRIVE guidelines.

### In utero electroporation (IUE) procedure

IUE using mice was performed as described previously with slight modifications^33,34^. Briefly, pregnant ICR mice were anesthetized, and the uterine horns were exposed. Approximately 1–2 μl of DNA solution was injected into the lateral ventricle of embryos using a pulled glass micropipette. Each embryo within its uterus was placed between a tweezer-type electrode (CUY650 P3, NEPA Gene). Square electric pulses (45 V, 50 ms) were passed 5 times at 1 s intervals using an electroporator (ECM 830, BTX). Care was taken to quickly place embryos back into the abdominal cavity to avoid temperature loss. The wall and skin of the abdominal cavity were sutured, and embryos were allowed to develop normally.

### Plasmids

Noggin was kindly provided by Dr. Daisuke Saito (Kyushu University) and was inserted into pCAG vector, yielding pCAG-noggin. sBMPR was kindly provided by Dr. Naoto Ueno (National Institute for Basic Biology, Okazaki, Japan) and was inserted into pCAG vector, yielding pCAG-sBMPR. The cDNA for mouse BMP7 was amplified using PCR with the following primers: 5′-ATTCTCGAGGCCACCATGCACGTGCGCTCGCTGC-3′ and 5′-AAGCGGCCGCCTAGTGGCAGCCACAGG-3′. The amplified fragment was digested with XhoI and NotI, and then subcloned into pCAG vector. pCAG-EGFP was described previously^35^. Plasmids were purified using the EndoFree Plasmid Maxi Kit (QIAGEN). To activate BMP signaling, a mixture of pCAG-EGFP (0.5 mg/ml) plus either pCAG-BMP7 or pCAG control plasmid (5 mg/ml) in PBS was used. To inhibit BMP signaling, a mixture of pCAG-EGFP (0.5 mg/ml) plus either pCAG-noggin, pCAG-sBMPR or pCAG control plasmid (5 mg/ml) was used. Fast Green solution was added at a final concentration of 0.3% to monitor the injection.

### Tissue preparation

Tissue sections were prepared as described previously^36,37^. Mice were deeply anesthetized and transcardially perfused with 4% paraformaldehyde (PFA)/PBS. Brains were dissected and post-fixed with an overnight immersion in 4% PFA/PBS, cryoprotected with 2-day immersion in 30% sucrose/PBS, and embedded in OCT compound. Coronal sections of 20–30 μm or 50 μm thickness were made using a cryostat.

### Immunohistochemistry

Immunohistochemistry was performed as described previously with slight modifications^38,39^. Briefly, free-floating sections were permeabilized with 0.3% Triton X-100/PBS, incubated overnight with primary antibodies in 2% bovine serum albumin (BSA)/PBS, and then washed with PBS 3 times for 10 min each. The sections were incubated with secondary antibodies and Hoechst 33342 in 2% BSA/PBS for 2 h, and then washed with PBS 3 times for 10 min each. The sections were mounted on slides with Mowiol (Sigma-Aldrich). Antibodies used were as follows: rabbit anti-aquaporin-4 (AQP4) antibody (Millipore), rabbit anti-glutamine synthetase (GS) antibody (Sigma-Aldrich), mouse anti-GS antibody (Roche), rabbit anti-NeuN antibody (Cell Signaling Technology), mouse CC1 antibody (Calbiochem), rabbit anti-phospho-Smad1/5/8 antibody (Cell Signaling Technology), rabbit anti-Cux1 antibody (Santa Cruz), rat anti-Ctip2 antibody (Abcam), donkey secondary antibodies conjugated with Cy3 (Jackson ImmunoResearch), donkey secondary antibodies conjugated with Alexa Fluor 647 (Molecular Probes) and donkey secondary antibodies conjugated with Alexa Fluor 488 (Molecular Probes).

### In situ hybridization

In situ hybridization was performed as described previously with slight modifications^37,40,41^. Briefly, sections of 20–30 μm thickness were incubated overnight with digoxigenin-labeled RNA probes in hybridization buffer (50% formamide, 5 × SSC, 5 × Denhardt's solution, 0.3 mg/ml yeast RNA, 0.1 mg/ml herring sperm DNA, and 1 mM dithiothreitol). The sections were then incubated with an alkaline phosphatase-conjugated anti-digoxigenin antibody (Roche) and were visualized using NBT/BCIP as substrates. In some experiments, the sections were then subjected to immunohistochemistry and Hoechst 33342 staining.

To make an RNA probe for mouse *AQP4*, the cDNA fragment for mouse AQP4 was amplified with RT-PCR using the following primers: 5'-AAAGAATTCTTTTGCTCGGTGTGGGAT-3' and 5'-AAAGAATTCGAAGCAAGAAACCCGCAA-3'. The amplified fragment was digested with EcoRI and then subcloned into pCRII-TOPO vector, and digoxigenin-labeled RNA probes were made. To make an RNA probe for mouse *BMP7*, digoxigenin-labeled RNA probes were made using pCAG-BMP7 plasmid.

### Microscopy

Epifluorescence microscopy was performed with BZ-9000 and BZ-X710 microscopes (KEYENCE).

### Quantification and statistical analysis

Coronal sections were stained with Hoechst 33342 plus either anti-GS antibody, anti-NeuN antibody, CC1 antibody, anti-AQP4 antibody or anti-phospho-Smad1/5/8 antibody. In some experiments, the sections were subjected to in situ hybridization for *AQP4*. Images captured using a BZ-9000 microscope were used for quantification. The background signal was removed by subtracting the average signal intensity of negative cells.

For cell counting, the numbers of immunopositive cells in columns with 200 μm width were manually counted using the “Cell Counter” tool of Image J software.

To measure the average intensities of AQP4 immunoreactivity, the regions in columns with 200 μm width corresponding to the GM or the WM were selected using the “Rectangle Selection” function of ImageJ. Average signal intensities in the regions were measured using the “ROI Manager” tool of ImageJ. To minimize variation in signal intensity depending on the position of each section, the average intensity of AQP4 on the electroporated side and that on the contralateral non-electroporated side of the cortex in the same brain section were measured, and the former was divided by the latter. Values in graphs are mean ± SD. An unpaired two-tailed Student's t-test was used to determine statistical significance. P values<0.05 were considered statistically significant.

## Supplementary Information

Supplementary Information 1.
