# Supplementary material for: BMP signaling alters aquaporin-4 expression in the mouse cerebral cortex
Source: Sci Rep. 2021 May 18;11:10540. doi: 10.1038/s41598-021-89997-5 (PMC8131757; doi:10.1038/s41598-021-89997-5)
Supplement: Supplementary file 1 — Supplementary Information 1. [file 41598_2021_89997_MOESM1_ESM.pdf]

Supplementary Materials

BMP signaling alters aquaporin-4 expression in the mouse cerebral cortex

Kazuya Morita, Naoyuki Matsumoto, Kengo Saito, Toshihide Hamabe-Horiike, Keishi Mizuguchi, Yohei Shinmyo and Hiroshi Kawasaki

Supplementary Figure 1

Supplementary Figure 2

Supplementary Figure 3

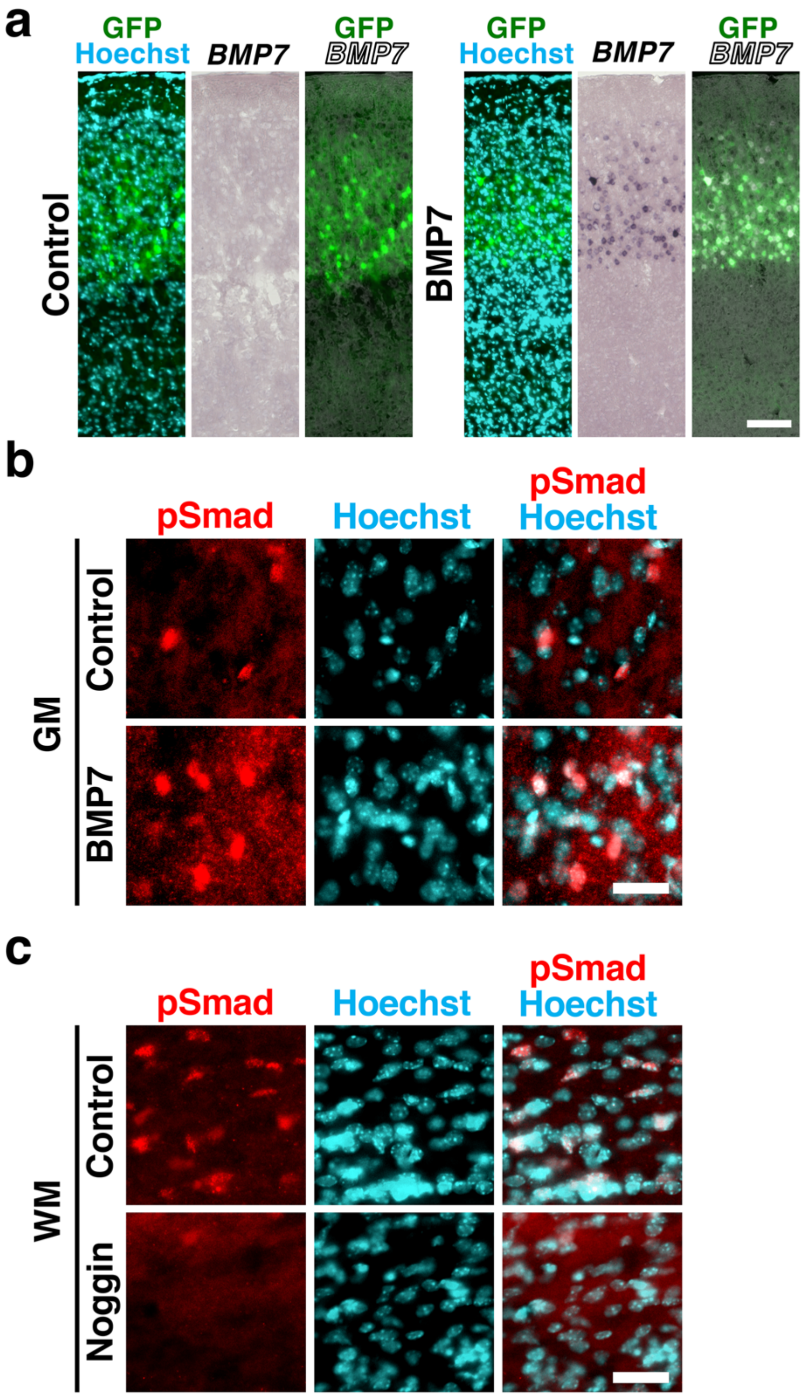

Supplementary Figure 1. The effects of BMP7 and noggin electroporation on BMP signaling

(a,b) pCAG-EGFP plus either pCAG-BMP7 or pCAG control vector was electroporated at E14, and the brains were dissected at P16. **(a)** Sections of the mouse cerebral cortex were subjected to *in situ* hybridization for *BMP7* and Hoechst 33342 staining. *BMP7*

mRNA signals were strongly induced by BMP7-expressing plasmids. (**b**) Sections were subjected to immunohistochemistry for pSmad. High magnification images of the gray matter are shown. Note that pSmad-positive cells were markedly increased by BMP7 electroporation. (**c**) pCAG-EGFP plus either pCAG-noggin or pCAG control vector was electroporated at E14, and the brains were dissected at P16. Sections were subjected to immunohistochemistry for pSmad. High magnification images of the white matter are shown. pSmad signals were strongly inhibited by noggin electroporation. GM, gray matter; WM, white matter. Scale bars = 100 μm **(a)** and 25 μm **(b,c)**.

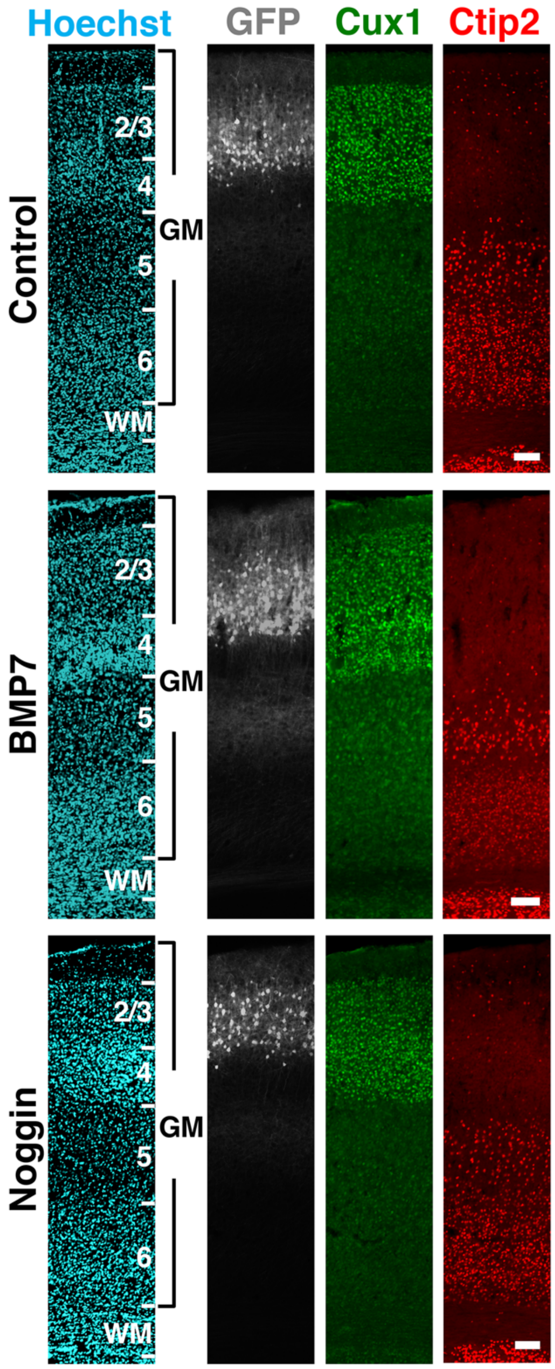

Supplementary Figure 2. Electroporation of BMP7 and noggin did not affect the layer structure of the cerebral cortex

pCAG-EGFP plus either pCAG-BMP7, pCAG-noggin or pCAG control vector was electroporated at E14, and the brains were dissected at P16. Sections of the cerebral cortex were subjected to Hoechst 33342 staining and immunostaining for Cux1 and Ctip2, which are expressed in layers 2-4 and in layers 5-6, respectively. Note that cortical layers were not affected by BMP7 and noggin. Numbers indicate the corresponding layers in the cerebral cortex. GM, gray matter; WM white matter. Scale bars = 100 μm.

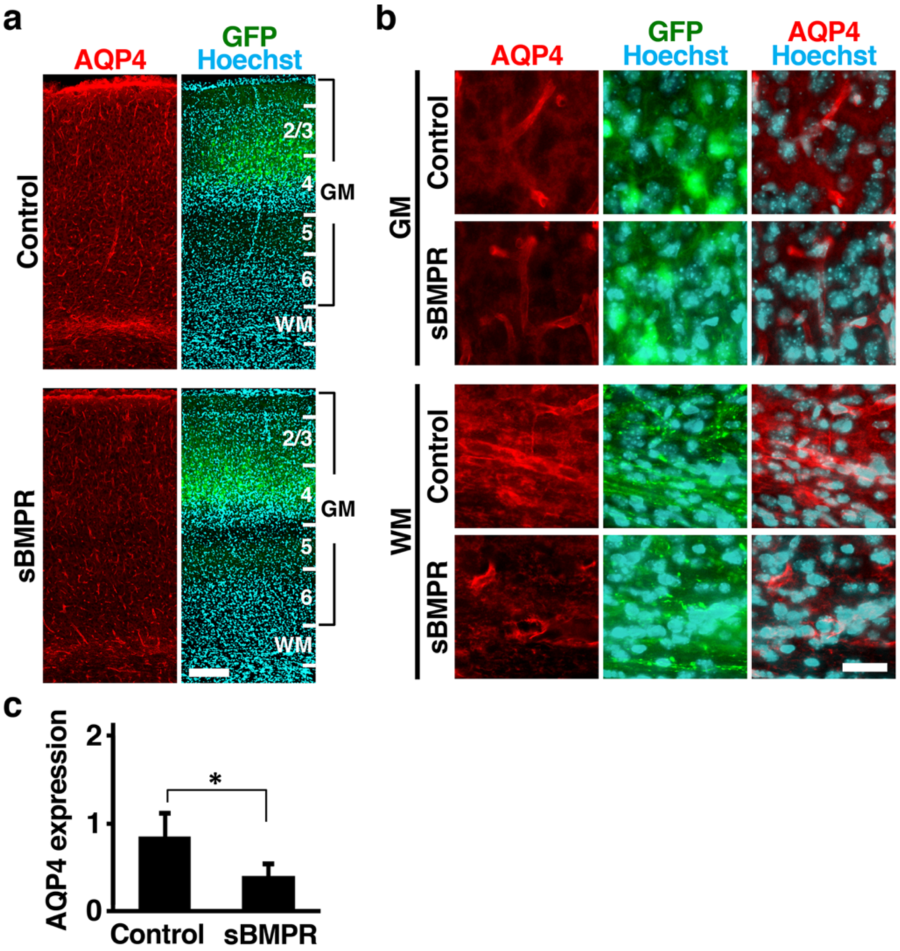

Supplementary Figure 3. Inhibition of BMP signaling reduces AQP4 levels in the developing mouse cerebral cortex

pCAG-EGFP plus either pCAG-sBMPR or pCAG control vector was electroporated at E14, and the brains were dissected at P16. **(a,b)** Coronal sections stained with anti-AQP4 antibody, anti-GFP antibody and Hoechst 33342. Low magnification images **(a)** and high magnification images **(b)** of the gray matter (GM) and the white matter (WM) are shown. The immunoreactivity of AQP4 in the white matter was markedly decreased by sBMPR. **(c)** Quantification of AQP4 immunoreactivity in the white matter. Inhibition of BMP signaling by sBMPR significantly decreased AQP4 immunoreactivity. n = 4 animals for each condition. Bars represent mean ± SD. *$p < 0.05$, Student's t-test. Numbers indicate the corresponding layers in the cerebral cortex. GM, gray matter; WM white matter. Scale bars = 200 μm **(a)** and 25 μm **(b)**.
